# Schistosomiasis in Malawi: a systematic review

**DOI:** 10.1186/s13071-014-0570-y

**Published:** 2014-12-10

**Authors:** Peter Makaula, John R Sadalaki, Adamson S Muula, Sekeleghe Kayuni, Samuel Jemu, Paul Bloch

**Affiliations:** Research for Health Environment and Development, Mangochi, Malawi; School of Public Health and Family Medicine, Department of Public Health, College of Medicine, University of Malawi, Chichiri, Blantyre 3 Malawi; Medical Aid Society of Malawi (MASM) Medi Clinics, Blantyre, Malawi; Ministry of Health, Capital City, Lilongwe 3, Malawi; Steno Health Promotion Center, Steno Diabetes Center, Niels Steensens Vej 8, Gentofte, DK-2820 Denmark

**Keywords:** Bilharzia, Biomphalaria, Bulinus, Schistosomiasis, Schistosoma haematobium, Schistosoma mansoni, Malawi

## Abstract

**Introduction:**

Schistosomiasis remains an important public health problem that undermines social and economic development in tropical regions of the world, mainly Sub-Saharan Africa. We are not aware of any systematic review of the literature of the epidemiology and transmission of schistosomiasis in Malawi since 1985. Therefore, we reviewed the current state of knowledge of schistosomiasis epidemiology and transmission in this country and identified knowledge gaps and relevant areas for future research and research governance.

**Methods:**

We conducted computer-aided literature searches of Medline, SCOPUS and Google Scholar using the keywords: “schistosomiasis”, “Bilharzia”, “Bulinus” and “Biomphalaria” in combination with “Malawi”. These searches were supplemented by iterative reviews of reference lists for relevant publications in peer reviewed international scientific journals or other media. The recovered documents were reviewed for their year of publication, location of field or laboratory work, authorship characteristics, ethics review, funding sources as well as their findings regarding parasite and intermediate host species, environmental aspects, geographical distribution, seasonality of transmission, and infection prevalence and intensities.

**Review:**

A total of 89 documents satisfied the inclusion criteria and were reviewed. Of these, 76 were published in international scientific journals, 68 were peer reviewed and 54 were original research studies. Most of the documents addressed urinary schistosomiasis and about two thirds of them dealt with the definitive host. Few documents addressed the parasites and the intermediate hosts. While urinary schistosomiasis occurs in most parts of Malawi, intestinal schistosomiasis mainly occurs in the central and southern highlands, Likoma Island and Lower Shire. Studies in selected communities estimated prevalence rates of up to 94.9% for *Schistosoma haematobium* and up to 67.0% for *Schistosoma mansoni* with considerable geographical variation. The main intermediate host species are *Bulinus globosus* and *Bulinus nyassanus* for urinary schistosomiasis and *Biomphalaria pfeifferi* for intestinal schistosomiasis*.* Seasonality of transmission tends to vary according to geographical, environmental, biological and behavioural factors.

**Conclusion:**

Transmission of schistosomiasis in Malawi appears to be highly focal, with considerable variation in space and time. Many locations have not been covered by epidemiological investigations and, thus, information on the transmission of schistosomiasis in Malawi remains fragmented. Functional infection risk assessment systems based on systematic investigations and surveillance are required for developing informed prevention and control strategies.

**Electronic supplementary material:**

The online version of this article (doi:10.1186/s13071-014-0570-y) contains supplementary material, which is available to authorized users.

## Introduction

Schistosomiasis ranks second only to malaria among the parasitic diseases affecting humans with regard to the number of people infected and the risk of becoming infected globally. The World Health Organization (WHO) recognizes schistosomiasis as one of the 17 neglected tropical diseases (NTDs), which are mostly persistent and prevalent in people and communities living in poverty and social exclusion, and jointly affects more than 1 billion people worldwide [1],[2]. It is estimated that schistosomiasis infects more than 200 million people globally and causes an annual loss of between 1.7 and 4.5 million disability adjusted life years (DALYs) [3]-[7]. A recent meta-analysis challenges these estimates and argues that a 0.5% disability weight assigned to schistosomiasis by the WHO is too low [8]. The authors noted that 2–15% disability weight is more realistic based on assessments of the different functional impairments of a person with schistosomiasis. It is estimated that more than 97% of all cases occur in Africa [9], and the highest prevalence estimates and infection intensities are usually found in school-age children, adolescents and young adults [10],[11] as well as in infants and pre-school children [12]. Schistosomiasis negatively impacts on children’s physical development and school performance, and the debilitation caused by untreated infections undermines social and economic development in heavily affected areas [13]-[16]. Schistosomiasis may also negatively affect the tourism industry of endemic countries as a result of fear among tourists and visitors of becoming infected [17]. The strategy recommended by WHO for the control of schistosomiasis includes preventive chemotherapy, which is an intervention that allows the regular and coordinated administration of quality-assured, safe, single dose medicines on a large scale [18].

In Malawi, urinary schistosomiasis caused by *Schistosoma haematobium* is highly prevalent in the lakeshore and southern region districts, while intestinal schistosomiasis caused by *Schistosoma mansoni* predominates on the central plains and in the northern region’s districts [19]. The National Schistosomiasis Control Programme (NSCP) estimates that 40-50% of the total Malawian population is at risk of becoming infected with schistosomiasis [20]. However, these national estimates appear to be derived from old surveys suffering from selection bias for high-risk schools. A national survey of primary school pupils in 2003 found large variation in the prevalence estimates of *S. haematobium* and *S. mansoni* infections among school children across the nation [20]. For *S. haematobium*, the estimates ranged from 0–43.1% with an average rate of 6.9%. For *S. mansoni*, the estimates ranged from 0–4.3%, with an average rate of 0.4%. The national survey used the urine filtration method to detect *S. haematobium* eggs in urine and the Kato-Katz smear method to detect *S. mansoni* eggs in stool. Single urine and stool samples were collected from every child and tested. These prevalence estimates were much lower than expected and it was therefore concluded that schistosomiasis transmission in Malawi is highly focal. This result implies that local estimates would be more reliable than national estimates in guiding the selection of control strategies to be implemented at district or sub-district level [5],[21]. Unfortunately, only few districts in Malawi, if any, have local prevalence estimates to guide the planning and implementation of prevention and control measures.

To our knowledge, this review is the third of a series of systematic literature reviews pertaining to schistosomiasis in Malawi. The first review, which was published by Blair in 1956 [22], revealed that *S. mansoni* infection was mainly observed in the northern district of Karonga and the extreme south, whereas *S. haematobium* infection was predominant in the lowlands. The second review, which was carried out by Teesdale in 1985 [19], indicated that *S. mansoni* infection was far more widespread than previously thought, predominantly in the central plateau, and that *S. haematobium* infection was highly prevalent in the southern region. Both infections occurred with moderate to high intensities along the lakeshore plain. The present paper systematically reviews the existing literature on schistosomiasis in Malawi, dating back to 1985, which was the last time a similar review was conducted in this country.

The main objective of this review is to update our understanding of the epidemiology and transmission of schistosomiasis in Malawi. The specific objectives are:

1. To characterize the existing literature on schistosomiasis in Malawi published or not published in scientific journals since 1985, with emphasis on year of publication, location of field or laboratory work, authorship, involved organizations, funding sources and ethics.
2. To summarize what is known about schistosomiasis in Malawi, in terms of parasite and intermediate host species, environmental aspects, geographical distribution, seasonality of occurrence and transmission, and infection prevalence and intensities.
3. To provide an update on existing schistosomiasis control efforts in Malawi with emphasis on policies, strategies and actions for integrated prevention and control of NTDs.
4. To identify knowledge gaps and research needs pertaining to the topic of schistosomiasis in Malawi.

## Methods

We carried out a systematic literature review of all online articles on schistosomiasis in Malawi since 1985 and published in the English language. As a first step, we undertook a computer-aided search of the electronic online databases Medline, SCOPUS and Google Scholar using the keywords: “schistosomiasis”, “Bilharzia”, “Bulinus” and “Biomphalaria” in combination with “Malawi”.

As a second step, we supplemented the searches by iterative reviews of reference lists of all included scientific publications for relevant additional literature available on the internet, including information websites. This additional literature included studies published in peer reviewed scientific journals, but also investigations available only in project reports, student dissertations and theses.

For the purpose of this review, a “research article” is defined as an original piece of scientific work published in an international and peer reviewed scientific journal; a “review article” is defined as a summarisation of existing knowledge and recent insights into specific research areas published in an international and peer reviewed scientific journal; a “project report” is defined as an original piece of scientific work, which has been published but not in an international and peer reviewed scientific journal; a “case report” is defined as a piece of scientific work, published in an international and peer reviewed journal, of one or more patients who have acquired schistosomiasis and received treatment; and a “letter to the editor” is a brief message written to an editor of an international and peer reviewed scientific journal, seeking to provide information or clarification on a certain topic of interest to the readers.

Inclusion and exclusion criteria for publications were defined prior to the review. Publications were included if reporting about findings from an area relating to schistosomiasis including laboratory work carried out in Malawi, or carried out outside of Malawi, but based on samples or specimens originating from Malawi since 1985, and if contributing factual knowledge about schistosomiasis epidemiology and transmission in Malawi. Publications were excluded if reporting on field or laboratory work on schistosomes based on samples or specimens originating from outside of Malawi, if field or laboratory work was carried out in Malawi before 1985, if only addressing theoretical issues, or if only expressing assumptions, beliefs, opinions, perceptions and attitudes about schistosomiasis.

**Figure 1:**
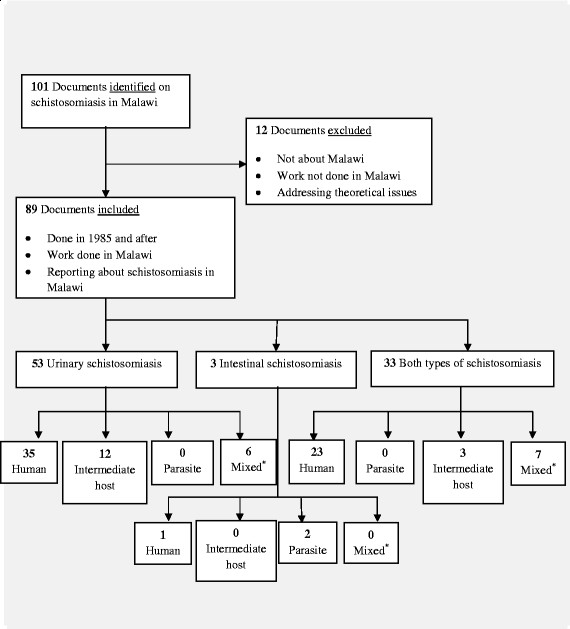
**Decision tree showing the number of included and excluded documents identified from online literature searches.** *Mixed = any document focusing on combination of (i) human, intermediate host and parasite; (ii) human and intermediate host; (iii) human and parasite or (iv) intermediate host and parasite.

As a third step, the documents were categorized into three groups according to the forms of schistosomiasis addressed (i.e. urinary schistosomiasis, intestinal schistosomiasis or a combination of the two). Each of these groups were further divided into four sub-groups according to the main focus of the study, namely: a) the definitive human host of the parasites, b) the intermediate host species of the parasites, c) the causative parasite species of the disease, e.g. studies that involved techniques for the detection of parasites in samples or specimens, or d) a mixture, i.e. any combination of two or all of the first focus areas of human, intermediate host and parasite. The decision tree for the inclusion or exclusion of documents and how they were assigned into these groups and sub-groups is shown in Figure 1.

Each of the included documents were reviewed for their basic characteristics including area of focus, year of publication, authorship, organizations involved, funding sources, location of field or laboratory work, adherence to ethics as well as its findings related to intermediate host and parasite species, geographical distribution, seasonality of occurrence and transmission, infection prevalence estimates and intensities, and relevance to tourism and other associated conditions.

## Review

### Literature on schistosomiasis in Malawi since 1985

The literature search yielded 101 documents responding to the applied keywords and published between 1985 and mid 2014. After further reviewing the literature and applying the inclusion and exclusion criteria, 12 documents failed to be included because they were not reporting on work carried out in or relating to Malawi. Thus, 89 documents met the inclusion criteria for this review. These 89 documents included 76 (85%) documents published in international scientific journals as research articles, review articles, case reports or letters to the editor; 68 (76%) were peer reviewed papers and 54 (61%) comprised original research articles. Characteristics of the reviewed documents are summarized in Table 1.

**Table 1:** **Characteristics of the included and reviewed documents**

Document type	Eligible documents	Published in scientific journal	Peer reviewed papers	Original research studies
Research article	54	47	47	54
Case report	18	18	18	0
Letter to the editor	9	7	0	0
Project report	4	1	0	0
Review article	4	3	3	0
**Total**	**89**	**76**	**68**	**54**

Of the 89 reviewed documents, 53 (60%) of them focused on urinary schistosomiasis, whereas 33 (37%) addressed both urinary and intestinal schistosomiasis. Only 3 (3%) documents focused exclusively on intestinal schistosomiasis.

The disparities in areas of focus were also observed in the sub-groups where, of the 53 documents in the urinary schistosomiasis group, 35 (66%) focused on humans, 12 (23%) focused on intermediate hosts and the remaining 6 (11%) focused on human, intermediate host and/or parasite. Of the 33 documents that focused on both types of schistosomiasis, 23 (70%) dealt with humans, 3 (9%) covered intermediate host(s), and 7 (21%) focused on human, intermediate host and/or parasite. Of the 3 documents in the intestinal schistosomiasis group, 1 (33%) focused on humans and 2 (67%) covered the parasite. Figure 1 summarizes the decision tree and how the documents have been classified according to their areas of focus.

### Year of publication, authorship, involved organizations, funding sources and ethics

**Figure 2:**
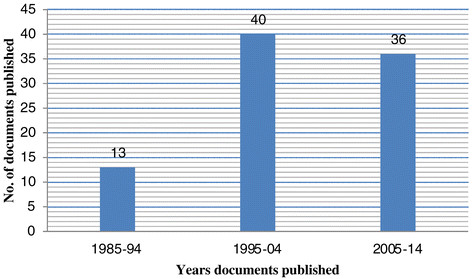
**Number of reviewed schistosomiasis related documents published between 1985 and mid 2014.**

All 89 documents included in this review were published over a period of 29 years between 1985 and mid 2014, which corresponds to an average publication rate of 3.0 papers per year. Further review revealed that publications on schistosomiasis in relation to Malawi have been increasing over time since 1985 (Figure 2). Scientific research and review articles dominated and represented 58 (65%) of the 89 documents. Moreover, 18 (20%) documents were case reports on foreign patients who were treated for schistosomiasis after visiting Malawi, 9 (10%) were letters to the editors of peer reviewed journals and 4 (5%) were project reports. We categorized 62 of the 89 documents as research articles, review articles or project reports. Of these documents, 26 (42%) were produced exclusively by research institutions based outside of Malawi; 5 (8%) were produced exclusively by local research institutions and 31 (50%) were produced by a combination of foreign and local research institutions (Table 2).

**Table 2:** **Characteristics of schistosomiasis related documents included in the review; The documents have been stratified by area of focus**

Serial No.	Ref. No.	Study area of focus/First author name and year	Total number of authors	Type of document	Originating country	Place(s) where study was done	Funding/Ethics^π^	Implementing agency^α^
		**A.** ***Urinary-human***						
1	[23]	Pullanikkatil, 2014	5	Research	Malawi	Zomba	Foreign/DHO	Local
2	[24]	Jourdan, 2013a	7	Research	Norway	Blantyre	Foreign/COM	Combined
3	[25]	Jourdan, 2013b	1	Research	Norway	Blantyre	Foreign/COM	Foreign
4	[26]	Logan, 2013	10	Case report	England	-	-	n/a
5	[27]	Chipeta, 2013	3	Research	Malawi	Chikhwawa	Foreign/COM	Local
6	[28]	Kjetland, 2012	3	Review	Norway	-	Foreign	Foreign
7	[29]	Blach, 2012	5	Research	Scotland	-	Foreign	Foreign
8	[30]	Kayuni, 2012	1	Research	Malawi	Mangochi	Foreign/MOH	Local
9	[31]	Yildirmak, 2012	4	Case report	Turkey	-	-	n/a
10	[32]	Kapito-Tembo, 2009	7	Research	Malawi	Blantyre	Foreign/UNC	Combined
11	[33]	Save the Children, 2008	1	Project report	USA	Mangochi	Foreign/MOH	Foreign
12	[34]	Yokota, 2007	13	Case report	Japan	-	-	n/a
13	[35]	Van Delft, 2007	4	Case report	Netherlands	-	-	n/a
14	[36]	Tsuboi, 2006	10	Case report	Japan	-	-	n/a
15	[37]	Moore, 2005	2	Letter	England	-	-	n/a
16	[38]	Tambo, 2004	7	Case report	Japan	-	-	n/a
17	[39]	Kitayama, 2004	14	Case report	Japan	-	-	n/a
18	[40]	Nicolas, 1998	5	Letter	France	-	-	n/a
19	[41]	Silwal, 1997	4	Case report	England	-	-	n/a
20	[42]	Kjetland, 1996	10	Research	Norway	Mangochi	Foreign/COM	Combined
21	[43]	Helling-Giese, 1996	12	Research	Germany	Mangochi	Foreign/COM	Combined
22	[44]	Poggensee, 1996	14	Research	Germany	Mangochi	Foreign/COM	Combined
23	[45]	Gundersen, 1996	9	Research	Norway	Mangochi	Foreign/COM	Combined
24	[46]	Cetron, 1996a	9	Research	USA	Lilongwe	Foreign/MOH	Combined
25	[47]	Potasman, 1996	4	Case report	Israel	-	-	n/a
26	[48]	TDR News, 1996	1	Letter	Switzerland	Mangochi	-	n/a
27	[49]	Hunt, 1995	1	Letter	England	Mangochi	-	n/a
28	[50]	Potasman, 1995	2	Case report	Israel	-	-	n/a
29	[51]	Herwaldt,1995	5	Case report	USA	-	-	n/a
30	[52]	Loefler, 1994	1	Letter	Kenya	Salima	-	n/a
31	[53]	Eulderink, 1994	4	Case report	Netherlands	-	-	n/a
32	[54]	Pugh, 1993	1	Letter	UAE	Mangochi	-	n/a
33	[55]	Whitworth, 1993	1	Letter	England	-	-	n/a
34	[56]	Wolfe, 1993	5	Research	Malawi	Lilongwe	Foreign/NO	Foreign
35	[57]	Russell, 1993	3	Letter	England	-	-	n/a
		**B.** ***Urinary – intermediate host***						
36	[58]	Madsen, 2012	2	Research	Denmark	Mangochi	Foreign	Foreign
37	[59]	Madsen, 2011	2	Research	Denmark	Mangochi	Foreign	Combined
38	[60]	Evers, 2011	3	Research	Denmark	Mangochi	Foreign	Foreign
39	[61]	Kubiriza, 2010	6	Research	Uganda	Mangochi	Foreign	Combined
40	[62]	Stauffer, 2008a	6	Research	USA	Mangochi	Foreign	Foreign
41	[63]	Lundeba, 2007	4	Research	Zambia	Mangochi	Foreign	Combined
42	[64]	Jørgensen, 2007	5	Research	Denmark	Mangochi	Foreign	Foreign
43	[65]	Lundeba, 2006	4	Research	Zambia	Mangochi	Foreign	Combined
44	[66]	Evers, 2006	4	Research	Denmark	Mangochi	Foreign	Foreign
45	[67]	Madsen, 2004	6	Research	Denmark	Mangochi	Foreign	Combined
46	[68]	Brown, 1996	6	Research	USA	Mangochi	Foreign	Combined
47	[69]	Chiotha, 1990	1	Research	USA	Mangochi	Foreign	Foreign
		***C. Urinary – parasite***						
		**D.** ***Urinary – mixed***						
48	[70]	Van Bocxlaer, 2014	3	Research	USA	Mangochi	Foreign	Foreign
49	[71]	Stauffer, 2013	3	Research	USA	Mangochi	Foreign	Foreign
50	[72]	Stauffer, 2008b	2	Project report	USA	Mangochi	Foreign/MOH	Foreign
51	[73]	Stauffer,2007	8	Research	USA	Mangochi	Foreign	Combined
52	[74]	Stauffer, 2006	8	Research	USA	Mangochi	Foreign	Combined
53	[75]	Madsen, 2001	5	Research	Denmark	Mangochi	Foreign	Combined
		**E.** ***Intestinal – human***						
54	[76]	Phiri, 2000	4	Research	Malawi	Blantyre/Chiradzulu	Local/COM	Combined
		***F. Intestinal – intermediate host***						
		**G.** ***Intestinal – parasite***						
55	[77]	Dacombe, 2007	7	Research	England	Karonga	Foreign/COM	Foreign
56	[78]	Chitsulo, 1990	3	Research	Malawi	Lilongwe	Foreign/MOH	Combined
		**H.** ***Intestinal – mixed***						
57	[79]	GAHI^¥^, 2002	1	Review	England	Malawi^¥^	Foreign/NO	Foreign
		**I.** ***Both – human***						
58	[19]	Teesdale, 1985	2	Review	Malawi	Malawi^§^	Foreign	Combined
59	[20]	Bowie, 2004	5	Research	Malawi	Malawi^μ^	Foreign/MOH	Combined
60	[80]	Berezowska, 2012	5	Research	Switzerland	Blantyre/Lilongwe	Foreign/MOH	Combined
61	[81]	Msyamboza, 2010	5	Research	Malawi	Southern Malawi^±^	Foreign/MOH	Combined
62	[82]	Redman, 2010	4	Review	Scotland	Malawi	Foreign	Foreign
63	[83]	Naus, 2003	5	Research	Netherlands	Blantyre	Foreign/COM	Combined
64	[84]	Gordon, 2003	6	Research	Malawi	Blantyre	Foreign/COM	Foreign
65	[85]	Kayuni, 2003	1	Research	Malawi	Malawi^Ω^	Local/COM	Local
66	[86]	Bloch, 2003	2	Research	Denmark	Likoma	Foreign/MOH	Combined
67	[87]	Jackson, 2003	3	Research	England	-	Foreign	Foreign
68	[88]	Randall, 2002	12	Research	England	Karonga	Foreign/COM	Combined
69	[89]	Trachtenberg, 2002	6	Research	USA	-	Foreign	Foreign
70	[90]	Salanitri, 2002	3	Case report	Australia	-	-	n/a
71	[91]	Waldman, 2001	4	Case report	England	-	-	n/a
72	[92]	Cooke, 1999	4	Case report	England	-	-	n/a
73	[93]	Hall, 1999	9	Research	England	Mangochi	Foreign/MOH	Foreign
74	[94]	MacLachlan, 1997	2	Research	Ireland	Zomba	Foreign/NO	Combined
75	[95]	Hipgrave, 1997	4	Case report	Australia	-	-	n/a
76	[96]	Jelinek, 1996	3	Case report	Germany	-	-	n/a
77	[97]	Ager, 1996	4	Research	Malawi	Mangochi	Foreign/NO	Combined
78	[98]	Wiselka, 1993	2	Case report	England	-	-	n/a
79	[99]	Ager, 1992	1	Research	Malawi	Mangochi	Foreign/NO	Foreign
80	[100]	Wolff, 1989	2	Project report	Malawi	Lilongwe/Zomba	Foreign/MOH	Combined
		**J.** ***Both – intermediate host***			USA	Mangochi	Foreign	Foreign
81	[101]	Stauffer, 2012	2	Research				
82	[102]	Phiri, 2002	1	Research	Malawi	Mangochi	Foreign	Local
83	[103]	Msukwa, 1997	1	Research	Malawi	Mangochi	Foreign	Foreign
		***K. Both – parasite***						
		**L.** ***Both – mixed***					Foreign/COM	
84	[104]	Poole, 2014	8	Research	England	Chikhwawa		Combined
85	[105]	Madsen, 2006	6	Research	Denmark	Mangochi	Foreign	Combined
86	[106]	Danida, 2000	1	Project report	Denmark	Mangochi	Foreign/MOH	Foreign
87	[107]	Stauffer, 1997	8	Research	USA	Mangochi	Foreign	Combined
88	[108]	Cetron,1996b	3	Letter	USA	Lilongwe	-	n/a
89	[109]	Wiselka, 1988	4	Research	England	Lilongwe	Foreign/NO	Foreign

^§^Blantyre, Chikhwawa, Chiradzulu, Chitipa, Dowa, Karonga, Kasungu, Lilongwe, Machinga, Mangochi, Mchinji, Mulanje, Mwanza, Mzimba, Nkhotakota, Nsanje, Ntchisi, Phalombe, Rumphi, Salima and Zomba.

^μ^Balaka, Chikhwawa, Chitipa, Dedza, Dowa, Kasungu, Lilongwe, Machinga, Mangochi, Mchinji, Mulanje, Mwanza, Mzimba, Nsanje, Ntcheu, Phalombe, Rumphi, Salima, Thyolo and Zomba.

^±^Blantyre, Chikhwawa, Chiradzulu, Mwanza, Phalombe and Thyolo.

^Ω^Balaka, Chikhwawa, Dedza, Karonga, Mangochi, Mchinji, Mzimba and Thyolo.

^¥^GAHI (Global Atlas of Helminth Infections): Chitipa, Karonga, Rumphi, Mzimba, Nkhata Bay, Kasungu, Mchinji, Ntchisi, Dowa, Lilongwe, Dedza, Salima, Ntcheu, Mangochi, Balaka, Machinga, Zomba, Blantyre, Chikhwawa, Nsanje and Mulanje.

^π^DHO=District Health Office; COM=College of Medicine; MOH=Ministry of Health; UNC=University of North Carolina; NO=Not Obtained.

^α^Implementing agency: Foreign=only foreign institution involved; Local=only Malawian institution involved; Combined=both foreign and Malawian institution involved; n/a=not applicable.

In total, 411 authors appeared on all 89 documents, giving a mean rate of 4.6 authors per document and a median of 4 authors (range 1–14) per document. Several authors appeared on more than one document. In total, there were 272 different authors involved in the publication of the 89 documents and 37 (14%) of these were Malawian nationals. Further analysis of authorship of the 89 reviewed documents showed that 10 (11%) had a Malawian as a first author and the remaining 79 (89%) had a non-Malawian as a first author. All 10 documents with a Malawian as a first author and 53 (67%) of the 79 documents with a non-Malawian as a first author originated from work carried out in Malawi. The remaining 26 (33%) documents with a non-Malawian as a first author originated from work carried out outside Malawi, such as laboratory studies based on samples and specimens originating from Malawi. Of the 62 documents categorized as research articles, review articles and project reports, 61 (98%) were funded by non-Malawi-based agencies, while only one student’s study was funded in Malawi, representing 2% of the documents.

Even though most of the reviewed publications derive from single-standing studies, four major groups of scientists have contributed comprehensively to publishing the findings from their research. These groups comprised several researchers who collaborated on specific research programs on schistosomiasis and related aspects in Malawi, namely 1) a group from Malawi, particularly from institutions within the University of Malawi, involved in publishing 17 (36%) scientific papers [6],[19],[20],[23],[26],[29],[31],[55],[75],[80],[83],[84],[96],[99],[101],[102] as first authors; 2) a group from USA involved in publishing 13 (28%) scientific papers [32],[45],[61],[67]-[73],[100],[106],[107] as first authors; 3) a group from Denmark involved in publishing 10 (21%) scientific papers [6],[57]-[59],[63],[66],[74],[85],[105] as first authors and 4) a group from Norway involved in publishing 7 (15%) scientific papers [24],[27],[41]-[44],[109] as first authors. It was not uncommon for a number of authors or institutions to collaborate, especially the groups from Denmark and the USA, and the groups from University of Malawi and Norway. In total, these four groups produced 47 (53%) of all documents reviewed. Of all the authors of the reviewed documents, Madsen from Denmark and Stauffer from USA were the most productive researchers, in terms of authorship and co-authorship of schistosomiasis related papers from Malawi. Of the 54 original research articles, these two authors appear individually or together on 19 (35%) of the articles.

All studies involving human subjects were reviewed for their ethical clearance with relevant authorities in Malawi or elsewhere prior to implementation. In total, 34 (63%) of the 54 original research studies involved human subjects. Of these, 14 (41%) mentioned that ethical clearance had been sought from the College of Medicine Research Ethics Committee (COMREC), 12 (35%) had sought ethical clearance from the Ministry of Health’s National Health Sciences Research Committee (NHSRC), 1 (3%) from the District Health Office and 1 (3%) from the Institutional Ethics Review Board of the University of North Carolina. The remaining 6 (18%) documents did not report on ethical clearance. The detailed characteristics of the reviewed documents are summarised in Table 2.

### Parasite and intermediate host species for human schistosomiasis in Malawi

There are two known species of parasites causing schistosomiasis in humans in Malawi, namely *Schistosoma haematobium* for the urinary form, and *Schistosoma mansoni* for the intestinal form. The review revealed that both species are present in Malawi. Moreover, two snail species are known to act as intermediate hosts for urinary schistosomiasis in Malawi, namely the well recognised host *Bulinus globosus* and the recently implicated *Bulinus nyassanus*[74], while *Biomphalaria pfeifferi* acts as intermediate host for intestinal schistosomiasis [108].

### Seasonality of transmission of schistosomiasis in Malawi

There are two possibilities for transmission of schistosomiasis within the Lake Malawi area, namely 1) by cercariae produced within the lake or 2) by cercariae transported into the lake by inflowing streams or rivers, or overflowing ponds close to the shores of the lake. *S. haematobium* appears to be the only schistosome transmitted in this lake. Transmission of schistosomiasis in the lake depends on factors such as human activity, wind and wave action, temperature, rainfall, lake level and density of intermediate hosts. Some studies have documented that transmission of urinary schistosomiasis in the southern part of Lake Malawi occurs mainly along sandy beaches where *B. nyassanus* is widely distributed or along relatively protected shore-lines such as harbours or calm bays where *B. globosus* is the primary intermediate host. Here, transmission has been documented to be low during the rainy season from December to April and part of the dry season from April to July. Transmission increases in the dry season from May and peaks in October, after which it decreases until the start of the rainy season around December when transmission stops. There may be local deviations in this pattern depending on the degree of wind and wave exposure of the shoreline. In the northern part of the lake, *B. nyassanus* is not found in shallower water possibly due to frequent strong wave action from June to August [58],[66],[71],[73],[101]. Seasonality of transmission in inland waters, beyond Lake Malawi, is caused by cercariae produced in rivers, streams, ponds and canals where *B. globosus* is the intermediate host; this seems particularly intense during the rainy season and for some time thereafter [66].

While much has been revealed regarding transmission of urinary schistosomiasis in Lake Malawi, not much has been documented about the seasonality of transmission of urinary schistosomiasis external to the lake as well as about transmission of intestinal schistosomiasis in Malawi in general. One study carried out in Mangochi between 2003 and 2007 reported that the intensity of transmission of *S. haematobium* in communities along the lakeshore was high as demonstrated by re-infection rates of 35% both in Cape Maclear and Msaka after offering mass treatment to school children in the previous year [71].

### Environmental aspects of schistosomiasis in Malawi

The impact of environmental changes affecting most classes of human pathogens has not spared schistosomiasis. The effects of unsafe water and lack of sanitation on the distribution and transmission of schistosomiasis in Malawi have been well documented [21],[23],[26],[29],[31],[75],[80],[103]. Several studies have addressed the effects of ecological and environmental changes on the distribution and transmission of schistosomiasis in Malawi [40],[57],[58],[65],[69],[70],[73],[100],[106]. It has been argued that increasing human population pressure and overfishing have decreased the densities of natural snail-eating fish species in the lake, which in turn has increased the densities of intermediate host species and, subsequently, the transmission of *S. haematobium*. Moreover, it has been argued that weather conditions, in terms of rain, wind, temperature, rainfall, lake level and wave action in the lake, have a strong influence on intermediate host densities and transmission of the infection [60],[66],[71],[74],[101].

### Geographical distribution of schistosomiasis in Malawi

**Figure 3:**
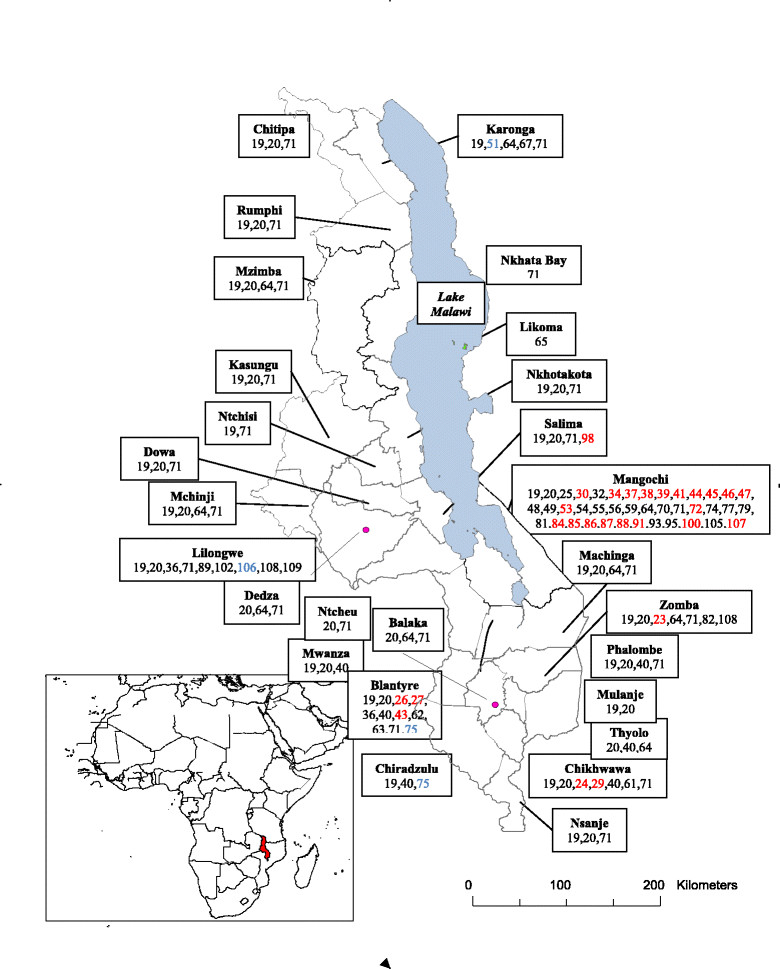
**Map of Malawi showing the geographical distribution of reviewed schistosomiasis studies.** The numbers refer to the reference list as follows: red numbers relate to urinary schistosomiasis; blue numbers relate to intestinal schistosomiasis and black numbers relate to both urinary and intestinal schistosomiasis.

The prevalence of schistosomiasis varies according to the distribution of intermediate hosts and schistosome parasites in water bodies and according to the behavioural patterns of humans. In order to determine the distribution of schistosomiasis in Malawi, the reviewed articles were linked to districts where initial research was carried out. From the 89 articles reviewed, 63 (71%) mentioned at least one specific district linked to their field or laboratory work. Figure 3 illustrates how these studies were distributed across Malawi. Mangochi was the most commonly mentioned district, with 40 references; 10 references each related to Lilongwe and Blantyre, 7 references related to Chikhwawa, 6 references related to Zomba, 4 references each related to Karonga, Mchinji, Salima and Mzimba, 3 references each related to Chiradzulu, Chitipa, Dowa, Kasungu, Machinga, Dedza, Balaka, Nsanje and Rumphi, 2 references each related to Mwanza, Phalombe, Thyolo, Mulanje, Ntcheu, Ntchisi and Nkhotakota, and one reference related to Likoma.

A total of 27 districts across Malawi were target sites for 33 studies dealing with the two forms of schistosomiasis during the period under review (Figure 3). Five districts comprising Mangochi, Blantyre, Chikhwawa, Zomba and Salima were target sites for 27 studies exclusively addressing urinary schistosomiasis, and 4 districts comprising Karonga, Lilongwe, Blantyre and Chiradzulu were target sites for 3 studies, which exclusively addressed intestinal schistosomiasis. One project collated and mapped (on its website) data related to the prevalence of *S. haematobium*, *S. mansoni*, schistosomiasis (both *S. haematobium* and *S. mansoni*), blood in urine, and soil transmitted helminths (STHs) produced by various surveys around the world [78]. This project successfully reported on the distributions of *S. haematobium*, *S. mansoni*, schistosomiasis and STHs in Malawi from 37 surveys carried out between 1982 and 2002 (and included in this review, if eligible). There was nothing reported from Malawi on blood in urine.

A total of 26 field surveys involving 25 districts were carried out to estimate infection prevalence rates at different times during the years between 1988 and mid 2014. For human infections with *S. haematobium*, 16 (62%) field surveys were carried out. All of these studies reported at least one infected individual, regardless of geographical area surveyed, and further observed infection prevalence rates ranging from 0.6% to 94.9% for *S. haematobium*. For human infections with *S. mansoni*, 3 (11%) field surveys were carried out in Mangochi, Blantyre and Chiradzulu, and the authors reported prevalence rates ranging from 0% to 2.1%. The remaining 7 (27%) surveys combined human infections with both *S. haematobium* and *S. mansoni*, and they reported infection prevalence rates ranging from 2.0% to 94.5% and from 0% to 67.0%, respectively, in the northern highlands, central lowlands, southern lowlands, urban areas, Chikhwawa and Chizumulu Island. These survey findings suggest that urinary schistosomiasis is more common and widely distributed in Malawi, and thus comprise a larger public health problem, than intestinal schistosomiasis [21],[23],[26],[31],[75],[78],[103].

Among the 22 field studies from where these findings originate, 11 (50%) were carried out in schools, 8 (36%) were carried out in communities and 3 (14%) were carried out in both schools and communities. The highest infection rates for *S. haematobium* were observed in the southern lowland districts of Mangochi, Phalombe and Chikhwawa, while the lowest infection rates were observed in the central highland district of Lilongwe and southern highland districts of Mwanza and Thyolo. For *S. mansoni*, the highest infection rates were observed in the central plains of Lilongwe, rural Chiradzulu and southern lowland district of Chikhwawa, and the lowest infection rates were found in the northern highlands, Likoma and Chizumulu Islands, central lowlands, urban districts of Mzuzu, Lilongwe and Blantyre, southern lakeshore district of Mangochi and in southern highlands. The details and a summary of the infection prevalence surveys carried out across Malawi during the period under review are shown in Table 3.

**Table 3:** **Schistosomiasis prevalence studies carried out in Malawi between 1988 and mid 2014**

Reference number	Year study was done	Place where study was done	Sample size	Target group examined	Diagnostic technique used	Infection species targeted	Prevalence (%)
[20]	2003	North high*	257	School children	Filtration	*S. haematobium*	7.4
[20]	2003	North high	257	School children	Kato Katz	*S. mansoni*	0
[20]	2003	Centre high^α^	242	School children	Filtration	*S. haematobium*	2.0
[20]	2003	Centre low^§^	291	School children	Filtration	*S. haematobium*	9.5
[20]	2003	Centre low	291	School children	Kato Katz	*S. mansoni*	0
[20]	2003	South high^Ω^	261	School children	Filtration	*S. haematobium*	3.2
[20]	2003	South high	261	School children	Kato Katz	*S. mansoni*	1.3
[20]	2003	South low^**±**^	297	School children	Filtration	*S. haematobium*	23.2
[20]	2003	South low	297	School children	Kato Katz	*S. mansoni*	0
[20]	2003	Urban^μ^	277	School children	Filtration	*S. haematobium*	3.4
[20]	2003	Urban	277	School children	Kato Katz	*S. mansoni*	0
[23]	2014	Zomba	483	School age children	Filtration	*S. haematobium*	34.0
[103]	2014	Chikhwawa	165	Mothers	Filtration	*S. haematobium*	45.1
[103]	2014	Chikhwawa	208	Pre-school children	Filtration	*S. haematobium*	17.7
[103]	2014	Chikhwawa	165	Mothers	Serology	*S. haematobium*	94.5
[103]	2014	Chikhwawa	208	Pre-school children	Serology	*S. haematobium*	49.5
[103]	2014	Chikhwawa	84	Mothers	Kato Katz	*S. mansoni*	21.5
[103]	2014	Chikhwawa	102	Pre-school children	Kato Katz	*S. mansoni*	0
[26]	2012	Chikhwawa	1642	Community based	Sedimentation	*S. haematobium*	14.2
[26]	2012	Chikhwawa	1642	Community based	Kato Katz	*S. mansoni*	4.3
[29]	2012	Mangochi	400	School children	Filtration	*S. haematobium*	12.5
[58]	1999	Mangochi	4324	Community based	Filtration	*S. haematobium*	39.0
[58]	1999	Mangochi	4131	School children	Filtration	*S. haematobium*	64.5
[80]	2008	Blantyre	14	Community based	Sedimentation	*S. haematobium*	28.6
[80]	2008	Chikhwawa	140	Community based	Sedimentation	*S. haematobium*	5.7
[80]	2008	Chiradzulu	178	Community based	Sedimentation	*S. haematobium*	5.6
[80]	2008	Mwanza	128	Community based	Sedimentation	*S. haematobium*	0.8
[80]	2008	Phalombe	39	Community based	Sedimentation	*S. haematobium*	94.9
[80]	2008	Thyolo	55	Community based	Sedimentation	*S. haematobium*	1.8
[31]	2006	Blantyre	1145	School children	Filtration	*S. haematobium*	10.4
[32]	1998	Mangochi	1084	School children	Filtration	*S. haematobium*	33.5
[32]	2007	Mangochi	1084	School children	Filtration	*S. haematobium*	4.5
[71]	2003	Mangochi	455	School children	Filtration	*S. haematobium*	36.5
[71]	2004	Mangochi	745	School children	Filtration	*S. haematobium*	35.7
[71]	2004	Mangochi	75	School children	Serology	*S. haematobium*	70.7
[71]	2005	Mangochi	1066	School children	Filtration	*S. haematobium*	21.9
[71]	2005	Mangochi	55	School children	Serology	*S. haematobium*	50.9
[71]	2006	Mangochi	1204	School children	Filtration	*S. haematobium*	36.4
[71]	2007	Mangochi	1100	School children	Filtration	*S. haematobium*	23.7
[85]	2003	Likoma	339	Community based	Filtration	*S. haematobium*	27.1
[85]	2003	Likoma	204	Community based	Kato Katz	*S. mansoni*	0.5
[85]	2003	Chizumulu	123	Community based	Filtration	*S. haematobium*	30.1
[85]	2003	Chizumulu	108	Community based	Kato Katz	*S. mansoni*	0
[85]	2006	Likoma	392	School children	Filtration	*S. haematobium*	30.0
[105]	1999	Mangochi	609	School children	Kato Katz	*S. mansoni*	0
[105]	1999	Mangochi	421	Community based	Kato Katz	*S. mansoni*	2.1
[105]	2000	Mangochi	4131	School children	Filtration	*S. haematobium*	23.8
[105]	2000	Mangochi	2316	Community based	Filtration	*S. haematobium*	17.2
[75]	2000	Blantyre	273	Village children	FEC^β^	*S. mansoni*	0
[75]	2000	Chiradzulu	280	Village children	FEC	*S. mansoni*	0.7
[45]	1993	Lilongwe	955	Expatriates	Serology	*S. haematobium*	32.0
[108]	1988	Lilongwe	165	Village children	Sedimentation	*S. haematobium*	0.6
[108]	1988	Lilongwe	165	Village children	Kato Katz	*S. mansoni*	67.0

*Chitipa, Rumphi and Mzimba; ^α^Ntcheu, Dedza, Dowa, Kasungu, Mchinji and Ntchisi; ^§^Salima and Nkhotakota; ^Ω^Phalombe, Mulanje, Thyolo, Mwanza and Balaka; ^±^Nsanje, Chikwawa, Mangochi, Machinga; ^μ^Mzuzu, Lilongwe and Blantyre; ^β^FEC = Formol Ether Concentration.

### Schistosomiasis prevention and control in Malawi

The history of schistosomiasis prevention and control efforts in Malawi dates back to the 1960s when the development of water resources prompted initiation of schistosomiasis control schemes [19]. The establishment of these schemes formed the basis for surveys estimating the magnitude and distribution of schistosomiasis in the country. The findings of these surveys led to the formation of the NSCP in the Ministry of Health. Between 1978 and 1990s, the NSCP conducted a number of nationwide prevalence surveys, confirming the widespread distribution of *S. haematobium* and *S. mansoni* in Malawi [19],[99],[108].

The NSCP has since been implementing centralized and vertical schistosomiasis prevention and control, characterized mainly by regular chemotherapy and health education in schools and villages [99]. In the new millennium, the NSCP has changed the strategy in several steps by decentralizing interventions, making efforts to bring on board STHs and further integrating control efforts with the NTDs as well as School Health and Nutrition (SHN) programs [9]. Moreover, there have been deliberate efforts to integrate schistosomiasis and STH prevention and control efforts within district implementation plans (DIPs) to strengthen local ownership and improve sustainability. Districts have been delivering treatments against both schistosomiasis and STHs in schools and communities during annual Child Health Day (CHD) campaigns.

An appraisal of 14 studies reporting from 21 surveys carried out to estimate the prevalence rates of schistosomiasis at different locations during the period between 1988 and 2014, revealed that the average prevalence rates of urinary schistosomiasis were 24.0% using the filtration method, 21.7% using the sedimentation method and 58.7% using serological methods, while intestinal schistosomiasis prevalence rates averaged 2.8% using the Kato Katz method and 0.7% using the Formol Ether Concentration method, as summarized in Table 4.

**Table 4:** **Summary of schistosomiasis prevalence surveys done in Malawi between 1988 and mid 2014**

Infection form	Where surveys were done			Total	Diagnostic technique used	No. of surveys*	Prevalence (%)	
	School	Community	Combined				Mean (range)	Median
Urinary	10	3	2	15	Filtration	16	23.7 (2.0-64.5)	23.7
					Sedimentation	3	19.0 (0.6-94.9)	5.7
					Serology	4	59.5 (32.0-94.5)	50.9
Intestinal	0	1	1	2	Kato Katz	6	7.4 (0–67.0)	0
					FEC^β^	1	0.7 (0–4.3)	0
Both	1	4	0	5	-	-	-	-
**Total**	**11**	**8**	**3**	**22**	-	-	-	-

*Some surveys used more than one diagnostic method; ^β^Formol Ether Concentration.

One study carried out in Mangochi in 1996 [41] investigated the occurrence of female genital schistosomiasis (FGS) amongst women infected with *S. haematobium*. It was found that 65% of the infected women had ova in the cervix, vagina or/and vulva by microscopy of genital biopsies; of which 76% were confirmed to have genital infection using colposcopic examinations. Associations have been observed between the incidence of FGS and HIV amongst women infected with *S. haematobium*; these women may be exposed to an additional risk of HIV transmission to or from their partners [27],[47].

### Schistosomiasis and tourism in Malawi

As one of Malawi’s main tourist destinations, Lake Malawi, has frequently been cited as a possible source of contracting schistosomiasis [45],[46],[48],[51],[53]-[56],[107]. While few documents mention any specific locations where tourists had contracted schistosomiasis, the popular tourist destination districts of Mangochi and Salima were mentioned as some of the places infested with the disease. However, due to lack of systematic monitoring of schistosomiasis transmission in Lake Malawi, and a lack of evidence about specific transmission sites, would-be visitors and tourists to Malawi have been warned about getting in touch with any natural water bodies whilst in the country [45],[55],[104]. Of the 89 reviewed documents, 22 (25%) reported on individuals who had developed signs and symptoms of schistosomiasis after returning from a visit to Malawi. These documents comprised 13 case reports and 9 letters to the editor written by non-Malawian authors [25],[30],[33]-[40],[46]-[56],[89]-[91],[94],[95],[97],[107].

## Discussion

We identified 89 documents on schistosomiasis in Malawi published since 1985, and there is a noticeable increase in the quantity of available literature as compared with the last review in 1985 [19], which mainly relied on unpublished survey data collected by the Ministry of Health across the country between 1976 and 1983. On average, 3 documents were produced per year since 1985, signifying an increase in interest in the schistosomiasis topic by researchers from both within and outside Malawi. Similarly, there was an increase in the number of both Malawian and non-Malawian authors involved in writing these documents. This finding suggests an increased capacity and ability to undertake research on schistosomiasis in Malawi.

The reviewed literature revealed that urinary schistosomiasis receives much more attention by the scientific community as compared with intestinal schistosomiasis. Thus, 97% of the reviewed documents included urinary schistosomiasis, whereas only 40% covered at least one aspect of intestinal schistosomiasis; more than one third included both forms of schistosomiasis and only 3 documents exclusively addressed intestinal schistosomiasis. This finding may be explained by the observation that urinary schistosomiasis is more common and widely distributed in Malawi and thus comprises a larger public health problem than intestinal schistosomiasis [20],[23],[26],[31],[103]. It may also be explained by the observation that urinary schistosomiasis is common in the major tourist destinations around Lake Malawi and thus receives substantial international attention [25],[28],[30],[36],[39],[45],[46],[55],[91],[97]; this may further explain the observation that the human aspects of urinary schistosomiasis receive more attention than other aspects of the infection [110]. For urinary schistosomiasis, more than two thirds of the reviewed documents covered aspects of the definitive human host, whereas less than a quarter of the documents addressed the intermediate host and none addressed the parasite itself. This apparent bias may relate to the limited resources available for schistosomiasis research. However, it might still be argued that non-human aspects of urinary (and intestinal) schistosomiasis need to be carefully studied to identify transmission sites and describe transmission intensities and patterns, seasonality and other determinants of infection of importance to prevention and control efforts [21].

Most of the articles under review were published between the years 1995 to 2010, including the period in the mid 90s when reporting began in the international media about the potential health hazard associated with visiting Malawi and about the linkages between water bodies, particularly Lake Malawi, and the scourge of schistosomiasis amongst tourists returning from visits to Malawi [36],[39],[45],[46],[48],[51],[53]-[56],[91],[97],[107]. Much of the research and publications that followed arose from reactions by the Government of Malawi and its international partners to the concerns raised by this international outcry on the schistosomiasis problem in the country. The response saw several international research institutions with or without partnering local institutions venturing into research in this field.

The reviewed literature revealed that almost half of the research institutions involved were foreign and that half of these operated through partnerships with local research institutions. It also revealed that almost all (98%) of the research carried out on schistosomiasis in Malawi was funded by foreign agencies. The typical arrangement was that local partner institutions contributed in kind support by providing personnel and equipment, while international partners provided funds. Both partners provided skills and expertise. Even though the schistosomiasis challenge is more of a local public health problem than a problem for foreigners, local funding for its research is almost non-existent. The numbers of Malawian first authors and their corresponding research institutions have not significantly increased during the period under review. This is somewhat paradoxical, considering that all our observations indicate that the concerns, subsequent research and ensued publications on schistosomiasis during the period under review were more foreign driven than local. Therefore, it would be appropriate for the Government of Malawi through relevant regulatory institutions to engage in discussions with international scientists and institutions about research capacity strengthening and collaboration in this important field.

There was almost no documentation produced exclusively by local researchers and research institutions on the risks and health consequences of contracting schistosomiasis in any water bodies in Malawi. It may be a lack of local attention to transmission dynamics and morbidity of infection that has attracted foreign research interests in this field. Most articles by foreign-based authors confirmed the existence of a risk of contracting schistosomiasis if exposed to Malawian water bodies, particularly Lake Malawi. This aspect has been discerned from published case studies involving some of the visitors who had been to Malawi and later developed symptoms of contracting the disease [25],[28],[30],[33],[35],[36],[38]-[40],[46],[51],[55],[81],[91]. Furthermore, we observed that research on schistosomiasis has not been evenly distributed geographically across the country. Some locations, such as Mangochi, Lilongwe, Blantyre and Chikhwawa, have been given higher priority by researchers than others. These biases make it difficult to draw any meaningful conclusions regarding the nationwide distribution and transmission of schistosomiasis in Malawi [111].

This review reaffirmed previous observations that *S. haematobium* for urinary schistosomiasis and *S. mansoni* for intestinal schistosomiasis are the dominant species of schistosomes infecting humans in Malawi. Moreover, important intermediate host species for infections with *S. haematobium* and *S. mansoni* are *Bulinus globosus* and *Biomphalaria pfeifferi,* respectively [61],[67],[72],[74],[78],[101],[103],[108]. However, another intermediate host species (*Bulinus nyassanus*) for *S. haematobium* was recently discovered [74]. This intermediate host appears to be responsible for much of the transmission that takes place in the lake waters along the beaches of Lake Malawi [74]. There is limited knowledge available on intermediate host species in most parts of Malawi other than Lake Malawi, because relatively few sites have undergone careful investigations, particularly of the intermediate host. As such, there is much to be done in terms of describing transmission dynamics on a wider geographical scale. One way forward is to institute widespread community-based transmission monitoring of the infection using trained environmental health workers or community volunteers across the country.

Prevalence studies carried out in selected locations nationwide between 1988 and 2014 revealed substantial variation in the prevalence of both *S. haematobium* and *S. mansoni*[20],[23],[26],[29],[31],[32],[45],[58],[71],[75],[80],[85],[103],[105],[108]. Some of this variation is caused by differences in distribution and transmission, and some is caused by differences in methodologies used in various studies. Methodological differences among studies carried out in different locations negate making meaningful comparisons of the findings. Nevertheless, prevalence estimates over the period under review appear to decline or stabilize in some locations, such as Mangochi, Likoma, Phalombe, Zomba and Chikhwawa; and some estimates have shown that the infection has remained low in locations such as Blantyre, Thyolo, Mwanza and Chiradzulu [20],[23],[26],[31],[32],[80],[99],[103],[105]. However, one study observed relatively high re-infection rates (of ~35%) with urinary schistosomiasis in selected lake-shore communities when re-examining the same children parasitologically 12 months after offering mass drug administration to high-risk groups [71]. This observation suggests that the transmission intensity at the community level has remained high and calls for long-term intervention and increased attention to optimized coverage in treatment campaigns.

Some studies from Malawi have addressed the relationship between schistosomiasis and associated conditions. One study observed that there is a high prevalence of female genital schistosomiasis (FGS) amongst women infected with *S. haematobium* and that this puts the women and their sexual partners at high risk of contracting HIV-infection. Consequently, this might have implications for marital and sexual life of the affected women [27],[41]. Another study carried out during the period under review has shed more light on several environmental factors such as human activity, wind and wave action, temperature, rainfall, lake level and density of intermediate hosts, which impact the distribution and prevalence of schistosomiasis in Malawi by influencing whether the life cycle of schistosomiasis can be completed or not [112]. Proper understanding of these relationships between schistosomiasis and associated conditions will go a long way in helping to inform future prevention and control strategies in Malawi.

Previously, schistosomiasis treatment, prevention and control program activities in Malawi through the NSCP were centralized and specific to a single disease. However, currently there have been deliberate efforts to decentralize and integrate schistosomiasis prevention and control efforts with those of STHs using the strategies recommended by WHO for the prevention and control of NTDs [1],[18],[113]. This is expected to provide synergistic effects of treatment efforts for both schistosomiasis and STHs within the overall context of NTDs [114]. Engaging district teams is expected to strengthen local ownership and improve sustainability. The NSCP operates with support from international partners, such as World Vision International, Merck, WHO, the Department for International Development (DfID), through the Integrated Control of Schistosomiasis in sub-Saharan Africa (ICOSA). Since 2009, the ICOSA project has been implementing a strategy to scale-up treatment by reaching over 5.6 million people from all over Malawi. Schistosomiasis and STHs treatment campaigns are carried out annually using Mass Drug Administration (MDA) offered by health workers through schools and communities. There is a need to further integrate these treatment campaigns with other ongoing programs, such as the expanded program for immunizations (EPI), maternal and child health (MCH), and other similar routes for distribution of drugs such as the WHO recommended community-directed intervention (CDI) approach, in order to reach other high risk groups, such as pre-school and out-of-school children [115].

In more than three of four studies reviewed, ethical clearance had been sought from the Ministry of Health’s NHSRC or the University of Malawi’s COMREC prior to execution of their research. The NHSRC, COMREC and the newly formed National Committee on Research in the Social Sciences and Humanities (NCRSH), which refer to the mother statutory entity, known as the National Commission for Science and Technology (NCST), have an overall mandate to promote, support, coordinate and regulate the development and application of research, science and technology in Malawi. However, from the present review, there appears to be some level of inconsistency about where researchers apply for ethical clearance for their research proposals in Malawi. Thus, 24% of the reviewed research studies appeared not to have sought any ethical clearance or to have obtained it from ineligible offices. There is a dire need for more coordination amongst these three entities under the ‘umbrella’ of the NCST to ensure that health research is not duplicative and is focused on areas that are in line with national priorities and also to ensure that schistosomiasis research, prevention and control are given much needed political priority as a public investment [9],[116].

There is a relationship between the widely publicized schistosomiasis problem and the crisis of the tourism industry in Malawi [25],[46],[51],[117]. The Malawi Government considers tourism to be a prime contributor to the country’s economy. This is outlined in the country’s strategic papers for attainment of the Millennium Development Goals and most recently in the Economic Recovery Plan (ERP) of 2012. For visitors who make it to the lake, there is no trustworthy information about the risk of transmission of schistosomiasis around lakeshore resorts and beyond. Therefore, there is an urgent and a strong local and national need for conducting systematic investigations of the seasonal transmission dynamics of urinary schistosomiasis in the lakeshore resorts around Lake Malawi and to build local institutional and human capacity in this highly specialized discipline.

While it is generally acknowledged that both forms of schistosomiasis are prevalent in Malawi, there is inadequate documentation about the transmission patterns at the local level to make sound judgements and statements about the risks of contracting infection in most communities and tourist resorts. Even where some research has been done, seasonality of transmission of schistosomiasis varies according to environmental, geographical, biological and behavioural factors [21],[112]. This situation has made it difficult for public health authorities to answer questions from members of the tourist sector, civil society organizations and other agencies regarding the safety of Malawian water bodies from contracting schistosomiasis. There is thus a need for a comprehensive strategy for nationwide monitoring of schistosomiasis distribution and transmission, which includes, among other things, the establishment of an efficient management and information system for schistosomiasis research and knowledge generated by key stakeholders, such as the relevant government departments, researchers, academia, tourist industry as well as international partners. This focus may be aligned with the Global Atlas of Helminth Infections’ ‘This Wormy World Project’ based at the London School of Hygiene and Tropical Medicine in the United Kingdom. [78].

This review has exposed the following knowledge gaps and possible areas for future research and research governance regarding schistosomiasis in Malawi:

1. *Distribution and transmission of urinary and intestinal schistosomiasis in Malawi*
  There is a need for a deliberate national research policy and strategy to ensure that schistosomiasis research is extended to the many locations not yet covered. This approach will create new and important knowledge on the geographical distribution and transmission of schistosomiasis in Malawi.
  There is a need for a deliberate national effort to ensure that research on the geographical distribution and transmission of intestinal schistosomiasis receives more attention. This will address current imbalances in the knowledge of disease epidemiology between urinary and intestinal schistosomiasis within Malawi.
  There is a need for more research focusing on the agents and intermediate hosts of *S. haematobium* and *S. mansoni*. This will address current imbalances in the level of knowledge about schistosomiasis intermediate hosts, agents and humans hosts in Malawi.
  There is further need for research to investigate the magnitude of the FGS problem in women infected with urinary schistosomiasis and how this relates to transmission of HIV between women and their sexual partners in Malawi.
2. *Efficacy and effectiveness of applied prevention and control strategies*
  There is a need to ensure that schistosomiasis research is integrated within the overall NSCP approach, as part of surveillance, monitoring and evaluation efforts. This will optimize research coordination and improve knowledge management on the efficacy and effectiveness of schistosomiasis prevention and control in Malawi.
  There is a need for adopting a multidisciplinary and integrated research approach by promoting the involvement of disciplines such as social, behavioural and biological sciences. This will contribute to a more detailed understanding of the disease and its determinants, and thus inform the development of more effective policies and strategies for the prevention and control of schistosomiasis in Malawi.
    There is a need for the NCST through its affiliates NHSRC, NCRSH and COMREC to provide necessary framework for discussing, coordinating and prioritizing health research in Malawi and to ensure that schistosomiasis research, prevention and control are given much needed political priority.
3. *Determinants of effectiveness of community-driven prevention and control*
  There is a need for more effectively engaging and empowering communities in the fight against schistosomiasis. This will support current NSCP decentralization processes whereby districts are empowered to integrate prevention and control activities in the District Implementation Plans.
4. *Establishment of comprehensive nationwide monitoring and surveillance of schistosomiasis*
  There is a need for setting up sentinel sites across the country for continued schistosomiasis transmission monitoring and surveillance. This will ensure continued availability of information on the distribution, locality and seasonality of schistosomiasis transmission in high-risk communities and tourist resorts. Moreover, it will involve training of local community members and health extension workers on how to collect, analyze and interpret data for decision making regarding prevention and control interventions at the local level in Malawi.
  There is a need for active involvement of the private sector. Such an approach would contribute to the sustainability of sentinel sites if they are operated on a demand-driven basis, as a service to clients, such as resort operators who have a strong interest in documenting transmission risks within the catchment areas of their resorts.

A strength of this review is that literature searches involved three comprehensive databases and followed up with iterative searches of bibliographies of identified documents for both published and unpublished reports. A possible limitation might be that we have relied on online searches and may thus have missed some hardcopy documents in libraries or offices. Moreover, the decision to exclude documents that were not written in English may have excluded some documents from being reviewed here. Nonetheless, in our opinion, this is a comprehensive systematic review, which provides valuable updated information about schistosomiasis in Malawi.

## Conclusion

This article reviews documentation about schistosomiasis in Malawi that has accumulated since 1985, which is the last time a similar review was published. While confirming the existence of the infection in Malawi, this documentation, however, has failed answering the frequently asked question regarding the safety of local water bodies from schistosomiasis. This limitation relates to poor geographical coverage of research efforts and a lack of deliberate policies and strategies for nationwide surveillance of transmission. While the capacity to conduct schistosomiasis research is available in Malawi, prevention and control efforts are suffering from limited funding and poor coordination amongst key stakeholders. Successes in schistosomiasis control in Malawi rely on intensified communication between researchers and public health professionals, and on increased commitment to surveillance and knowledge management as well as to prevention and control efforts supported by informed policies, strategies and administrative structures at the national, district and community levels.

## Authors’ original submitted files for images

Below are the links to the authors’ original submitted files for images. Authors’ original file for figure 1 Authors’ original file for figure 2 Authors’ original file for figure 3
