# Supplementary figures and images for: Schistosomiasis in Malawi: a systematic review
Source: Parasit Vectors. 2014 Dec 10;7:570. doi: 10.1186/s13071-014-0570-y (PMC4288699; doi:10.1186/s13071-014-0570-y)

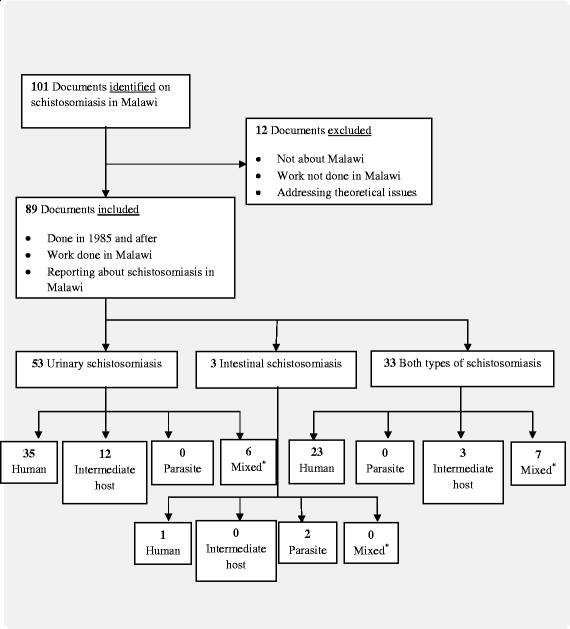

Supplement: Supplementary file 1 — Authors’ original file for figure 1 [file 13071_2014_570_MOESM1_ESM.gif]

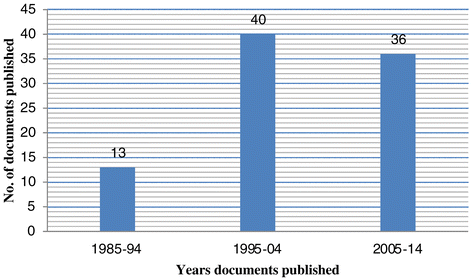

Supplement: Supplementary file 2 — Authors’ original file for figure 2 [file 13071_2014_570_MOESM2_ESM.gif]

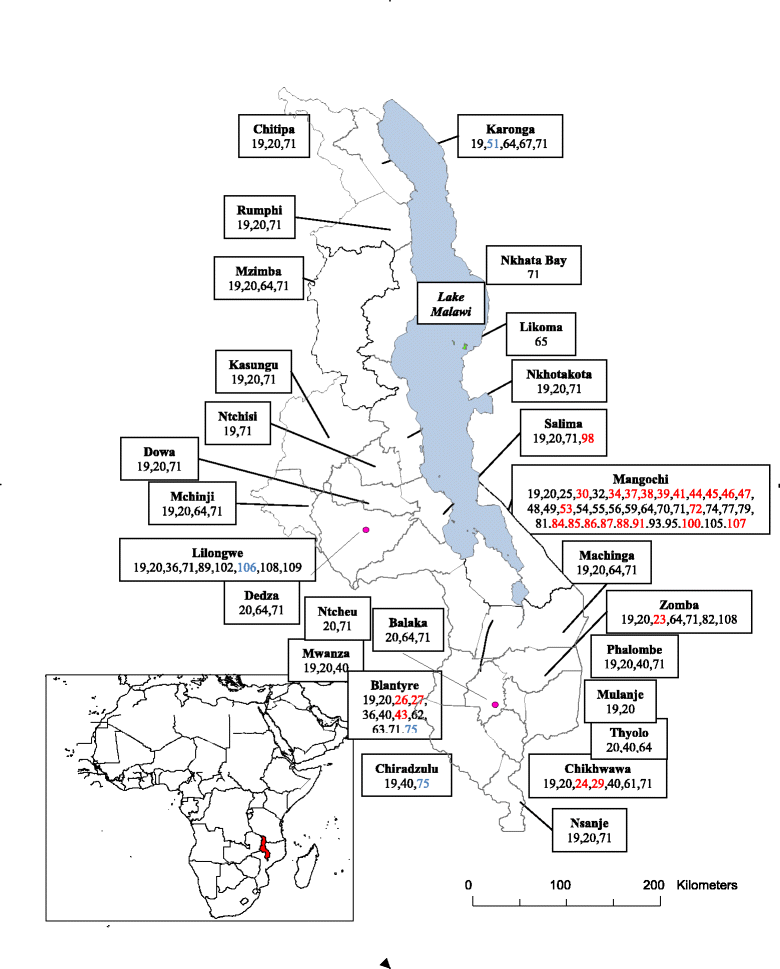

Supplement: Supplementary file 3 — Authors’ original file for figure 3 [file 13071_2014_570_MOESM3_ESM.gif]
